# Utilization of Preventive Measures for Glucocorticoid-Induced Osteoporosis among Veterans with Inflammatory Bowel Disease

**DOI:** 10.1155/2013/862312

**Published:** 2013-04-21

**Authors:** Aikaterini Thanou, Tauseef Ali, Omar Haq, Sindhu Kaitha, Jordan Morton, Stavros Stavrakis, Mary Beth Humphrey

**Affiliations:** ^1^Arthritis and Clinical Immunology Research Program Oklahoma Medical Research Foundation, 825 NE 13th Street, MS 22, Oklahoma City, OK 73104, USA; ^2^Inflammatory Bowel Disease Center, 825 NE 10th Street, Oklahoma City, OK 73014, USA; ^3^Department of Medicine, University of Oklahoma Health Sciences Center, P.O. Box 26901, WB1140, Oklahoma City, OK 73126, USA; ^4^Oklahoma City Veterans' Affairs Medical Center, 921 NE 13th Street, Oklahoma City, OK 73104, USA

## Abstract

*Purpose*. We examined current osteoporosis prevention practices in patients with inflammatory bowel disease (IBD) on chronic steroid using the 2003 American Gastroenterological Association guidelines as standard of care. *Methods*. We identified all IBD patients followed at the Oklahoma City VA Medical Center from January 2003 to December 2010, who had been on daily oral steroids (prednisone ≥5 mg or budesonide ≥6 mg) for ≥3 consecutive months. Associations of calcium and vitamin D (vitD) prescribing and bone mineral density (BMD) testing with patient characteristics were examined by logistic regression. *Results*. Sixty-three of 384 consecutive patients met inclusion criteria. Among 86 steroid courses, calcium and vitD were concurrently prescribed in 46%, and BMD was tested in 30%. There was no association of demographic and clinical characteristics with calcium/vitD prescribing and BMD testing. By multivariate analysis, steroid initiation after 2006, compared to before 2006, was associated with a significant increase in calcium (OR = 3.17 and *P* = 0.02) and vitD (OR = 2.96 and *P* = 0.02) prescribing and BMD testing (OR = 4.63 and *P* = 0.004). *Conclusions*. We observed a low, yet increasing, adherence to osteoporosis prevention guidelines in IBD since 2003, which highlights the need for continued physician education to enhance guideline awareness and implementation.

## 1. Introduction

Patients with inflammatory bowel disease (IBD) have an increased prevalence of osteoporosis ranging from 17% to 41% [1], whereas low bone mass has been shown to translate into an up to a 40% higher relative risk of fractures in this population [2]. The etiology of osteoporosis in IBD is multifactorial, with risk factors including malnutrition, calcium and vitamin D malabsorption, immobilization, corticosteroid use, and the underlying inflammatory state [3]. Steroid-induced osteoporosis is a specific concern in IBD patients because it is associated with rapid loss of bone mass soon after steroid initiation and translates into an increased fracture risk, independent of that determined by age and bone mineral density (BMD) alone [4, 5]. Despite the availability of therapies to reduce fracture risk, patients on long-term steroids often do not receive any intervention to prevent or treat osteoporosis [6, 7]. Guidelines for osteoporosis screening and prevention in IBD patients on chronic corticosteroids were published by the American Gastroenterological Association (AGA) in 2003 [1].

According to the AGA guidelines, IBD patients with any additional risk factors for osteoporosis, for example, prolonged corticosteroid use (≥3 consecutive months or recurrent courses), history of low trauma fracture, postmenopausal state for females, and hypogonadism or age above 50 for males, should be screened for osteoporosis with BMD testing. For patients with normal bone density (T score >−1), basic preventive measures are advised, such as steroid avoidance, (that is, the use of the minimum steroid dose for the least duration), smoking cessation, limitation of excessive alcohol intake, regular weight bearing exercise, and adequate calcium and vitamin D intake, along with periodic BMD monitoring. In individuals with osteopenia (T score <−1 but >−2.5), additional treatment with bisphosphonates should be considered. Finally, patients with osteoporosis (T score <−2.5) should be treated with bisphosphonates in addition to basic preventive measures. Implementation of the AGA guidelines in patients with IBD has previously been shown to be low [8, 9], and trends of adherence to these guidelines over time remain unclear. We aimed to examine current practices on osteoporosis prevention and management in patients with IBD on chronic steroid therapy using the AGA guidelines as standard of care.

## 2. Methods

Through an electronic search of the computerized patient record system (CPRS), we identified all consecutive patients with an ICD9-CM code compatible with Crohn's disease (555.0, 555.1, 555.2, and 555.9) or ulcerative colitis (556.0, 556.1 to 556.6, 556.8, and 556.9) who had an encounter at the gastroenterology (GI) clinic or the endoscopy laboratory of the Oklahoma City Veterans' Affairs (VA) hospital from January 2003 to December 2010. We subsequently identified all patients on daily oral steroids (prednisone ≥5 mg daily or budesonide ≥6 mg daily) for ≥3 consecutive months dispensed by the VA pharmacy from January 2003 to June 2011. Topical glucocorticoids and methylprednisolone dose packs were excluded. Prescription fill dates and days of supply per refill were used to assess duration of each steroid course. The average daily prednisone equivalent dose was calculated by dividing the cumulative dose dispensed (number of pills × pill strength) by the number of fill days prescribed. Only steroid courses prescribed by GI, rheumatology, the emergency room, or the patients' primary care providers for documented disease flare were included to minimize prescription bias (e.g., prescription for other indications). 

The demographic and clinical characteristics of patients, including age, gender, race, type and duration of IBD, steroid type (prednisone and budesonide), duration of steroid use, year of steroid initiation, body mass index (BMI) values, and serum albumin levels at the time of steroid treatment were collected. We identified all prescriptions of calcium and vitamin D dispensed by the VA pharmacy within the same interval of treatment with long-term steroids. We also collected information on BMD scans performed during steroid treatment or within 12 months prior to steroid initiation. The study was approved by the institutional review board of the University of Oklahoma Health Sciences Center.

## 3. Statistical Analysis

Continuous variables are presented as mean ± standard deviation (SD) or median and interquartile rage (IQR), as appropriate. Categorical variables are presented as percentages. Logistic regression was used to identify factors significantly associated with prescription of calcium, vitamin D, and BMD scans. Variables found to be associated by univariate analysis were entered into a multivariate model to identify independent predictors of prescribing of calcium and vitamin D, and BMD testing. Odds ratios (OR) with 95% confidence intervals (95% CI) for the respective variables were calculated, with statistical significance declared at *P* < 0.05. All statistical analyses were performed using SAS version 9.2 (Cary, NC).

## 4. Results

### 4.1. Patient Population

The baseline characteristics of the patient cohort are summarized in Table 1. Three hundred eighty four consecutive patients with IBD were identified over 7 years of observation, 72 of whom received daily oral steroids for ≥3 consecutive months. Nine of those patients were excluded because of steroids being prescribed for cancer, chronic obstructive pulmonary disease (COPD), or as posttransplantation drug therapy. Thus, 63 patients were included in the final analysis. There were 61 males, 57 Caucasian, 4 African American, and 2 Caucasian females. The mean patient age at the time of steroid administration was 55.0 ± 13.9 years, with 22 individuals below age 50 years. Twenty seven patients had Crohn's disease, and 36 had ulcerative colitis. The median duration of IBD at initiation of long-term steroids was 2.0 years (IQR 1 to 8 years).

A total of 86 steroid courses (69 prednisone and 17 budesonide) of ≥3 month duration were prescribed in 63 patients. Fifty one individuals received long-term prednisone and 9 budesonide, whereas 3 received separate courses with prednisone and budesonide. The median daily dose of prednisone was 15 mg (IQR 9 to 25 mg). Budesonide was administered at a dose of 9 mg in all but one patient who received 6 mg. Steroids were administered for a median of 6 consecutive months (IQR 4 to 12). Thirty eight (44.2%) courses were prescribed by GI providers, 10 (11.6%) by rheumatologists, 18 (20.9%) by primary care, and 4 (4.6%) by the Emergency Department or the inpatient service, whereas prescribing location was not specified in 16 (18.6%) of the steroid courses. Fourteen steroid courses were prescribed in 10 individuals with BMI <21 kg/m^2^, and 52 courses were prescribed in 37 individuals with serum albumin <3.5 g/dL at one or more occasions during steroid treatment. No association was observed between low BMI and concurrent hypoalbuminemia (*P* = 0.78).

Calcium, vitamin D, and bisphosphonates were concurrently prescribed in 40 (46.5%), 39 (45.3%), and 23 (26.7%) of steroid courses, respectively. One or more osteoporosis preventive treatments were concurrently prescribed in 49 (56.9%) of the courses. BMD was tested during steroid treatment or within 12 months prior to steroid initiation in 26 (30.2%) steroid courses. BMD scans associated with 11 steroid courses in 11 patients showed osteopenia, and calcium/vitamin D and bisphosphonates were administered in 9 (81.8%) and 8 (72.7%) of those courses, respectively.

### 4.2. Predictors of Osteoporosis Preventive Measures

Neither the age at IBD diagnosis nor the age at steroid initiation was associated with prescribing of calcium and vitamin D, and BMD testing. Likewise, gender, race, type and duration of IBD, and duration of steroid treatment were not found to be associated with prescribing of calcium and vitamin D, and BMD testing (*P* > 0.05 for each comparison by univariate analysis) (Table 2). However, calcium prescribing was associated with higher BMI, the year of steroid initiation, and performance of BMD, irrespective of results. Similarly, vitamin D prescribing was associated with the year of steroid initiation and performance of BMD. However, when patients with abnormal BMD results were excluded, the association of calcium (*P* = 0.07) and vitamin D (*P* = 0.17) with performance of BMD was lost, indicating that the association was driven by calcium and vitamin D prescribed for osteoporosis or osteopenia. The year of steroid initiation, type of steroid (prednisone or budesonide), and hypoalbuminemia were also associated with BMD testing by univariate analysis (Table 2). 

Of the variables associated with calcium and vitamin D prescribing by univariate analysis, the year of steroid initiation modeled as a dichotomous variable remained significant in multivariate analysis and was the strongest independent predictor of preventive osteoporosis measures (Table 3). Specifically, steroid initiation after the median year (2006) was associated with a more than two-fold increase in calcium (OR = 3.17, 95% CI: 1.19–8.37, and *P* = 0.02) and vitamin D (OR = 2.96, 95% CI: 1.16–7.58, and *P* = 0.02) prescribing. Similarly, a later year of steroid initiation was the only independent predictor of BMD testing, both when the year of initiation was modeled as a continuous variable (OR = 1.35, 95% CI: 1.09–1.68, and *P* = 0.007) and as a categorical variable. In the latter analysis, steroid initiation after 2006 was associated with a more than four-fold increase in BMD testing (OR = 4.63, 95% CI: 1.63–13.17, and *P* = 0.004).

## 5. Discussion

In this retrospective cohort study of IBD patients on long-term steroids, 46% or less received concurrent calcium or vitamin D supplementation or BMD testing. This observation is in accordance with previous studies of patients with IBD [8, 9] and male veterans on long-term steroids for other indications [6, 7]. These results may reflect unfamiliarity or conflict in interpretation of published guidelines, time constraints, and failure to recall indications to institute therapy among other reasons [7]. The belief that IBD should be the focus of the visit and that osteoporosis should be managed by another physician is an additional barrier previously detected among GI providers [10]. Despite our relatively small patient sample, prescribing of calcium, vitamin D, and bisphosphonates was nontheless high among those on long-term steroids, who were diagnosed with osteopenia or osteoporosis, reflecting increasing awareness of the necessity of treatment once a diagnosis of osteoporosis or osteopenia is established in this population. 

Although adherence to the AGA guidelines for osteoporosis prevention was overall poor in our cohort, we observed a favorable trend of increasing adherence to the guidelines since 2003. Calcium supplementation and BMD testing respectively increased, on average, by 21% and 39% yearly from 2003 to 2010. When considering the year of steroid initiation as a dichotomous variable, prescribing of calcium and vitamin D, and BMD testing were 3.17, 2.96, and 4.63 times more likely after 2006 compared to before 2006. Such a temporal tend has been previously demonstrated for the ACR guidelines on prevention of glucocorticoid-induced osteoporosis in diverse populations on long-term steroids [11], though not for the AGA guidelines in patients with IBD. Considering the absence of systematic educational or administrative interventions targeting the patients or their providers in our institution, increased implementation of preventive measures is probably multifactorial, reflecting heightened awareness of osteoporosis risk by both patients and their providers, among other reasons. No variations by prescribing specialty were found, despite previous observations of higher or lower prescribing rates in subspecialties [6, 11]. Importantly, these results highlight the need for continued education of physicians in the respective specialties in order to enhance the awareness and the implementation of guidelines for prevention of osteoporosis.

Prescribers and patients may avoid supplementary calcium and vitamin D or bisphosphonates for a variety of reasons, including adequate dietary intake by food sources, gastrointestinal intolerance, or particular contraindications (e.g., hypercalciuria or nephrolithiasis for calcium supplements, renal insufficiency, esophageal dysmotility, or peptic ulcers for bisphosphonates, and adequate serum vitamin D levels for vitamin D supplements). Associated medical conditions might also function as competing comorbidities deterring physicians from prescribing osteoporosis preventive treatment [6]. Gastrointestinal disorders have been particularly associated with an 85% reduction in the odds of osteoporosis prophylaxis and may be of particular importance in this population [6]. In support of this hypothesis, there was less prescribing of calcium supplements for patients on long-term steroids with low BMI (OR = 0.26, 95% CI: 0.07–1.00, and *P* = 0.05), an indicator of poor nutrition as well as independent risk factor of osteoporosis and fracture risk [12]. 

Although basic preventive measures and screening for osteoporosis are universally recommended, the role of BMD testing and bisphosphonate treatment in younger men and premenopausal women on steroids is less well established. Low bone mass in this age group can be reversible after steroid cessation and is not necessarily associated with an increased fracture risk [5, 13], whereas bisphosphonate treatment can be expensive and potentially harmful in this population. Even in older individuals, traditional (age, smoking, and hypogonadism) and IBD specific risk factors for osteoporosis (e.g., vitamin D deficiency, malnutrition, and the underlying inflammatory state) and any prior fragility fractures need to be weighed in before deciding on bisphosphonate therapy. The WHO fracture risk predictor model (FRAX) designed to incorporate such factors in a comprehensive assessment tool has shown promising preliminary results in patients with IBD [14], though further testing and validation is needed. We did not observe an association of age with prescribing of calcium, vitamin D, and bisphosphonates or with BMD testing in this cohort, an observation possibly biased by inadequate assessment of all contributing factors influencing the actual fracture risk for each individual.

Budesonide, an oral steroid with extensive first pass metabolism through the liver and low systemic bioavailability, is considered to have less corticosteroid related effects on bone metabolism. Budesonide was associated with better preservation of bone mass compared to prednisolone in cortricosteroid naïve patients with ileocecal Crohn's disease [15]. We observed no difference in prescribing of calcium or vitamin D between prednisone and budesonide. The lower likelihood of BMD testing with prednisone compared to budesonide courses (OR = 0.29, 95% CI: 0.10–0.87, and *P* = 0.03) probably reflects other unidentified confounders. 

*Limitations*. Data on steroid, calcium, vitamin D, and bisphosphonate prescriptions filled at the VA pharmacy are robust, since eligible patients obtain the majority of their medications through the VA. Nonetheless, some patients may have elected to get their medications though non-VA pharmacies or to use over-the-counter calcium and vitamin D supplements. It is similarly possible that BMD scans were ordered by the physicians but eventually not obtained or were obtained outside the VA in patients with additional insurance coverage. Although provisions are in place for entering non-VA and over-the-counter medications or results from outside studies into the VA database, such entries were rare in our cohort, likely causing underestimation of utilization of osteoporosis preventing treatments. Socioeconomic parameters determining benefit eligibility and copayments were not accounted in this study though these have been well documented to affect prescribing patterns [16]. Previous studies showed higher rates of medication dispensing to African Americans through the VA, possibly due to increased access to pharmacy benefits [6], but the small numbers of non-Caucasians in our study did not allow comparisons with race. Similarly, the small number of women included did not allow examination of variability due to gender. The unique characteristics of the VA patients may also limit the generalizability of our results to the overall IBD population. 

## 6. Conclusions

In summary, we demonstrated that implementation of preventive measure in our population of veterans with IBD treated with long-term steroids remains low but demonstrates a favorable trend over time. Our results indicate the need for continuing education of providers and patients with IBD on osteoporosis prevention and treatment. Ongoing research on genetic and disease specific risk factors for osteoporosis in patients with IBD may additionally allow updating of the current guidelines to incorporate a comprehensive assessment of osteoporosis risk possibly including the FRAX tool [14].

## Figures and Tables

**Table 1:** Baseline characteristics.

Variable	
Gender (male/female)	61/2
Race (Caucasian/African American)	57/4
Mean age at steroid initiation ± SD (years)	55.0 ± 13.9
>age 50 years (%)	22
Type of IBD (Crohn's disease/ulcerative colitis)	27/36
Median IBD duration at steroid initiation (IQR) (years)	2.0 (1–8)
Number of steroid courses	86
Prednisone	69
Budesonide	17
Median daily prednisone dose (IQR) (mg)	15 (9–25)
Median course duration (IQR) (months)	6 (4–19)
Courses by prescribing providers (%)	
GI	38 (44.2)
Rheumatology	10 (11.6)
Primary care	18 (20.9)
ER/inpatient service	4 (4.6)
Unspecified	16 (18.6)
Albumin < 3.5 g/dL (%)	52 (60.4)
BMI < 21 kg/m^2^ (%)	14 (16.3)
Calcium (%)	40 (46.5)
Vitamin D (%)	39 (45.3)
Bisphosphonates (%)	23 (26.7)
Any treatment (%)	49 (56.9)
BMD scans	26
Osteopenia	11
Osteoporosis	1

**Table 2:** Associations of clinical variables with calcium and vitamin D prescribing, and BMD testing by univariate analysis.

Variable	Calcium			Vitamin D			BMD		
	*P* value	OR	95% CI	*P* value	OR	95% CI	*P* value	OR	95% CI
Age at steroid initiation									
Continuous (per year)	0.36			0.69			0.65		
≥50 versus <50	0.82			0.21			0.38		
Age at IBD diagnosis (per year)	0.33			0.22			0.39		
Gender (males versus females)	0.98			0.98			0.55		
Race (Caucasian versus African American)	0.88			0.85			0.39		
Diagnosis (Crohn's disease versus ulcerative colitis)	0.85			0.63			0.10		
IBD duration (per year)	0.76			0.83			0.07		
Steroids (budesonide versus prednisone)	0.96			0.69			**0.03**	** 0.29**	0.10–0.87
Steroid duration (per month)	0.42			0.37			0.49		
Prednisone dose (per mg)	0.11			0.13			0.06		
Location	0.64			0.82			0.15		
BMI <21 kg/m^2^	**0.05**	**0.26**	0.07–1.00	0.06			0.44		
Albumin <3.5 g/dL	0.33	****		0.11			**0.04**	**2.92**	1.03–8.28
Year of steroid initiation		****							
≥2006 versus <2006	**0.002**	**4.25**	1.72–10.48	**0.003**	**3.81**	1.55–9.34	**0.002**	**5.04**	1.83–13.92
Continuous (per year)	**0.04**	**1.21**	1.01–1.45	0.10			**0.002**	**1.39**	1.13–1.72
BMD	**0.008**	**2.42**	1.26–4.65	**0.01**	**2.27**	1.21–4.27	NA		
BMD (excluding abnormal exams)	0.07			0.17			NA		

**Table 3:** Associations of clinical variables with calcium and vitamin D prescribing, and BMD testing by multivariate analysis.

Variable	Calcium			Vitamin D			BMD		
	*P* value	OR	95% CI	*P* value	OR	95% CI	*P* value	OR	95% CI
Steroids (budesonide versus prednisone)							0.31		
BMI <21 kg/m^2^	0.06								
Albumin <3.5 g/dL							0.09		
Year of steroid initiation									
≥2006 versus <2006	**0.02**	**3.17**	1.19–8.37	**0.02**	**2.96**	1.16–7.58	**0.004**	**4.63**	1.63–13.17
Continuous (per year)							**0.007**	**1.35**	1.09–1.68
BMD	**0.04**	**2.04**	1.02–4.08	0.07			NA		
