# Corrigendum to “Utilization of Preventive Measures for Glucocorticoid-Induced Osteoporosis among Veterans with Inflammatory Bowel Disease”

**DOI:** 10.1155/2017/2365840

**Published:** 2017-09-18

**Authors:** Aikaterini Thanou, Tauseef Ali, Omar Haq, Sindhu Kaitha, Jordan Morton, Stavros Stavrakis, Mary Beth Humphrey

**Affiliations:** ^1^Arthritis and Clinical Immunology Research Program Oklahoma Medical Research Foundation, 825 NE 13th Street, MS 22, Oklahoma City, OK 73104, USA; ^2^Inflammatory Bowel Disease Center, 825 NE 10th Street, Oklahoma City, OK 73014, USA; ^3^Department of Medicine, University of Oklahoma Health Sciences Center, P.O. Box 26901, WB1140, Oklahoma City, OK 73126, USA; ^4^Oklahoma City Veterans' Affairs Medical Center, 921 NE 13th Street, Oklahoma City, OK 73104, USA

In the article titled “Utilization of Preventive Measures for Glucocorticoid-Induced Osteoporosis among Veterans with Inflammatory Bowel Disease” [1], there was an error regarding the FRAX® tool, which should be clarified as follows.

The article notes, “the WHO fracture risk predictor model (FRAX) designed to incorporate such factors in a comprehensive assessment tool has shown promising preliminary results in patients with IBD”. However, the World Health Organization (WHO) did not develop, test, or endorse the FRAX tool or its recommendations [2]. The metabolic bone disease unit at the University of Sheffield that developed FRAX was a WHO Collaborating Centre from 1991 to 2010, but treatment guidelines must undergo a formal process before they can be endorsed by the WHO.
